# Serotonergic and immunomodulatory properties of the psychobiotic candidate *Bacteroides finegoldii* UO.H1052 and its extracellular vesicles

**DOI:** 10.1128/aem.00891-25

**Published:** 2025-08-21

**Authors:** Basit Yousuf, Galal Ali Esmail, Nazila Nazemof, Nour Elhouda Bouhlel, Zoran Minic, Riadh Hammami

**Affiliations:** 1 NuGut Research Platform, School of Nutrition Sciences, Faculty of Health Sciences, University of Ottawa 6363, Ottawa, Canada; 2 John L. Holmes Mass Spectrometry Facility, Faculty of Science, University of Ottawa 151175, Ottawa, Canada; 3 Department of Biochemistry, Microbiology and Immunology, Faculty of Medicine, University of Ottawa 151173, Ottawa, Canada; University of Illinois Urbana-Champaign, Urbana, Illinois, USA

**Keywords:** *Bacteroides finegoldii*, probiotics, extracellular vesicles, immunomodulatory properties, serotonin-secretagogue capacity, neurotransmitters, psychobiotics, gut-brain axis

## Abstract

*Bacteroides finegoldii* UO.H1052, a human gut commensal, was evaluated for its potential psychobiotic and immunomodulatory properties. Whole-genome analysis confirmed the absence of virulence factors, plasmids, and antibiotic-resistance genes. Metabolomic profiling of cell-free supernatants (CFSs) and extracellular vesicle (EV) postbiotics revealed a high- and medium-dependent production of neuroactive metabolites, including γ-aminobutyric acid, tryptophan, tyrosine, and tyramine, as well as physiologically relevant levels of short-chain fatty acids, such as acetate, propionate, and butyrate. Functionally, CFS enhanced epithelial barrier integrity by increasing transepithelial electrical resistance and mitigating LPS-induced disruption in Caco2/HT29 monolayers without cytotoxic effects. Both CFS and EVs exhibited immunomodulatory properties, characterized by elevated *Il-10*/*Tnf-α* ratios under basal conditions and significant suppression of *Tnf-α* expression in LPS-stimulated RAW 264.7 macrophages. Notably, CFS and EVs increased tryptophan hydroxylase 1 (*Tph1*) gene expression in enterochromaffin RIN14B cells by 6.6- and 3.2-fold, respectively, suggesting enhanced serotonergic activity. These findings highlight *B. finegoldii* UO.H1052 as a promising next-generation psychobiotic candidate with neuroactive, barrier-protective, and immunoregulatory properties, supporting its potential for gut-brain axis modulation.

**IMPORTANCE:**

Emerging evidence supports the critical role of the gut microbiota in modulating host neurophysiology and immune function via the gut-brain axis. Here, we present a comprehensive characterization of *Bacteroides finegoldii* UO.H1052, a human gut commensal that exhibits promising psychobiotic attributes, including the production of neuroactive compounds and extracellular vesicles (EVs) with immunoregulatory and serotonin-inducing properties. The strain exhibits a favorable safety profile, with no detected virulence factors or transmissible antibiotic resistance. Importantly, cell-free supernatants and EVs enhanced epithelial barrier integrity, modulated pro- and anti-inflammatory cytokine responses, and significantly upregulated the expression of *Tph1*, a key enzyme in serotonin biosynthesis. These findings underscore the potential of *B. finegoldii* UO.H1052 as a next-generation psychobiotic candidate and highlight EVs as effective postbiotic mediators of host-microbe communication. This study advances the understanding of *Bacteroides*-derived psychobiotics and provides a foundation for their development in modulating gut-brain and immune pathways relevant to neuroinflammatory and gastrointestinal disorders.

## INTRODUCTION

Probiotics are live microorganisms that confer health benefits when consumed in adequate amounts (1). Traditionally, probiotic research has primarily focused on *Lactobacillus* and *Bifidobacterium* genera due to their generally recognized as safe (GRAS) status and widespread use in food products (2). However, advances in microbiome research and a rapidly expanding global probiotic market (projected to exceed USD 132.78 billion in 2029) have prompted interest in next-generation probiotics (NGPs) (3). These emerging candidates, typically sourced from the human microbiota, offer unique and potentially targeted health benefits. Members of the phylum Bacteroidota are emerging as promising candidates for NGP (4, 5).

*Bacteroides* species are dominant anaerobic bacteria in the human gut and play pivotal roles in host physiology through diverse metabolic, immunomodulatory, and signaling activities. These signaling functions include the production of bioactive molecules such as short-chain fatty acids (SCFAs), polysaccharide A, and neurotransmitter precursors, which may interact with host receptors to influence immune responses and gut-brain communication (6). Several strains, such as *Bacteroides ovatus*, *Bacteroides salyersiae*, and *Bacteroides fragilis*, have been reported to exhibit strain-specific beneficial properties, with *B. ovatus* showing potential anti-inflammatory activity (7, 8), *B. salyersiae* implicated in colitis modulation (9), and *B. fragilis* associated with mucosal recovery (10). Likewise, *Bacteroides dorei* has been reported to influence host physiology by metabolizing cholesterol (11) and has also been associated with enhanced efficacy of COVID-19 vaccination, potentially by promoting Th1 immune responses and dendritic cell maturation (12). Similarly, *Bacteroides vulgatus* has been implicated in the regulation of host sugar intake via the gut-liver-brain axis through pantothenate signaling (13) . Additionally, γ-aminobutyric acid (GABA)-producing *Bacteroides* strains have been associated with constipation relief and gut-brain axis modulation (14). The phylum Bacteroidota has also been linked to neuroactive functions and brain health (15). For example, oral administration of *B. fragilis* restored gut barrier integrity and improved behavioral outcomes in a mouse model of autism spectrum disorder (16). Higher Bacteroidota abundance is also associated with elevated serotonin and myoinositol levels, key molecules in gut-brain signaling, and inversely linked with depression-related brain signatures (15, 17). Notably, *Bacteroides uniformis* has demonstrated stress-alleviating effects by modulating SCFAs and amino acid metabolism (18).

Growing evidence points to the pivotal role of microbial extracellular vesicles (EVs), nanosized membrane-derived structures, in mediating microbial-host communication (19). Bacteroidota EVs exhibit anti-inflammatory activity *in vitro* and *in vivo*, particularly in models of inflammatory bowel disease (20–22). For instance, *B. fragilis* EVs promote IL-10 secretion via a TLR2 receptor-dependent pathway (23), while *Bacteroides thetaiotaomicron* EVs trigger IL-10 production via the TLR2-MyD88 axis (24), in which TLR2 activation initiates a downstream signaling cascade that requires the adaptor protein MyD88 to mediate cytokine production. These EVs cross the mucus layer to modulate intestinal inflammation (21) and traverse the blood-brain barrier (BBB) to be internalized by microglia and immature neuronal cells without inducing significant inflammation (25).

Our previous findings underscored strain-specific production of neuroactive compounds, including GABA, by *Bacteroides* spp. (26). We identified *Bacteroides finegoldii* UO.H1052 as a strain with potent neuroactive metabolite production, including GABA-enriched EVs capable of modulating host immune responses (26, 27). In this study, we present a comprehensive functional characterization of *B. finegoldii* UO.H1052 by integrating genomic, metabolomic, and cellular assays to evaluate its psychobiotic potential. The production of SCFAs and neurotransmitter precursors in CFS and EVs is reported, along with key probiotic features. The capacity of the strain to enhance gut epithelial barrier integrity, modulate cytokine expression, and upregulate serotonin biosynthesis is also evaluated.

## RESULTS

### Comparative genomic analysis of plasmids, antibiotic resistance, and virulence factors in Bacteroidota strains

We assessed the safety of a selection of 18 Bacteroidota strains previously isolated in our laboratory (26), with a focus on the genomic features associated with pathogenicity and antibiotic resistance. None of the genomes harbored the *B. fragilis* toxin (*bft*) gene. The plasmids were predicted in all strains of the genus *Phocaeicola* and five strains of *Bacteroides,* but none of the strains of *Parabacteroides* (Table 1). Antimicrobial resistance (AMR) analysis of these plasmids revealed that only those located in *Phocaeicola dorei* UO.H1033, *Bacteroides stercoris* UO.H1035, *B. stercoris* UO.H1039, and *Bacteroides zhangwenhongii* UO.H1054 harbored AMR genes. The gene *cfxA3,* which confers resistance to β-lactam antibiotics, was found in the plasmid of the UO.H1033 strain. Plasmids of UO.H1035, UO.H1039, and UO.H1054 possessed the same *tetQ* gene, which mediates tetracycline resistance and is typically associated with conjugative transposons. Toxin-antitoxin (TA) systems, which play a crucial role in the stability and persistence of plasmids through new generations, were identified in all strains containing plasmids (Table 1).

**TABLE 1:** Identification of genomic features related to the safety of strains with neuroactive properties

Strain	Virulence gene *bft^b^*	Plasmid detection	AMR (plasmid)		TA systems
			*tetQ*	*cfxA3*	
***B. finegoldii* UO.H1052^*a*^**	−	−	−	−	−
*Bacteroides cellulosilyticus* UO.H1027	−	−	−	−	−
*B. cellulosilyticus* UO.H1030	−	−	−	−	−
*Bacteroides faecis* UO.H1051	−	−	−	−	−
*Bacteroides caccae* UO.H2003	−	+	−	−	+
*B. zhangwenhongii* UO.H1054	−	+	+	−	+
*B. ovatus* UO.H1053	−	+	−	−	+
*B. stercoris* UO.H1035	−	+	+	−	+
*B. stercoris* UO.H1039	−	+	+	−	+
*B. stercoris* UO.H2001	−	−	−	−	−
*B. uniformis* UO.H1043	−	−	−	−	−
*Parabacteroides johnsonii* UO.H1047	−	−	−	−	−
*P. johnsonii* UO.H1049	−	−	−	−	−
*Phocaeicola massiliensis* UO.H1001	−	+	−	−	+
*P. massiliensis* UO.H1004	−	+	−	−	+
*Phocaeicola vulgatus* UO.H1015	−	+	−	−	+
*P. vulgatus* UO.H1016	−	+	−	−	+
*P. dorei* UO.H1033	−	+	−	+	+

^*a*^ Bold indicates the strain selected for functional evaluation in this study.

^*b*^ − means absence.

### Genome characteristics and safety of *Bacteroides finegoldii* UO.H1052

#### Overall genomic insights

**Fig 1:**
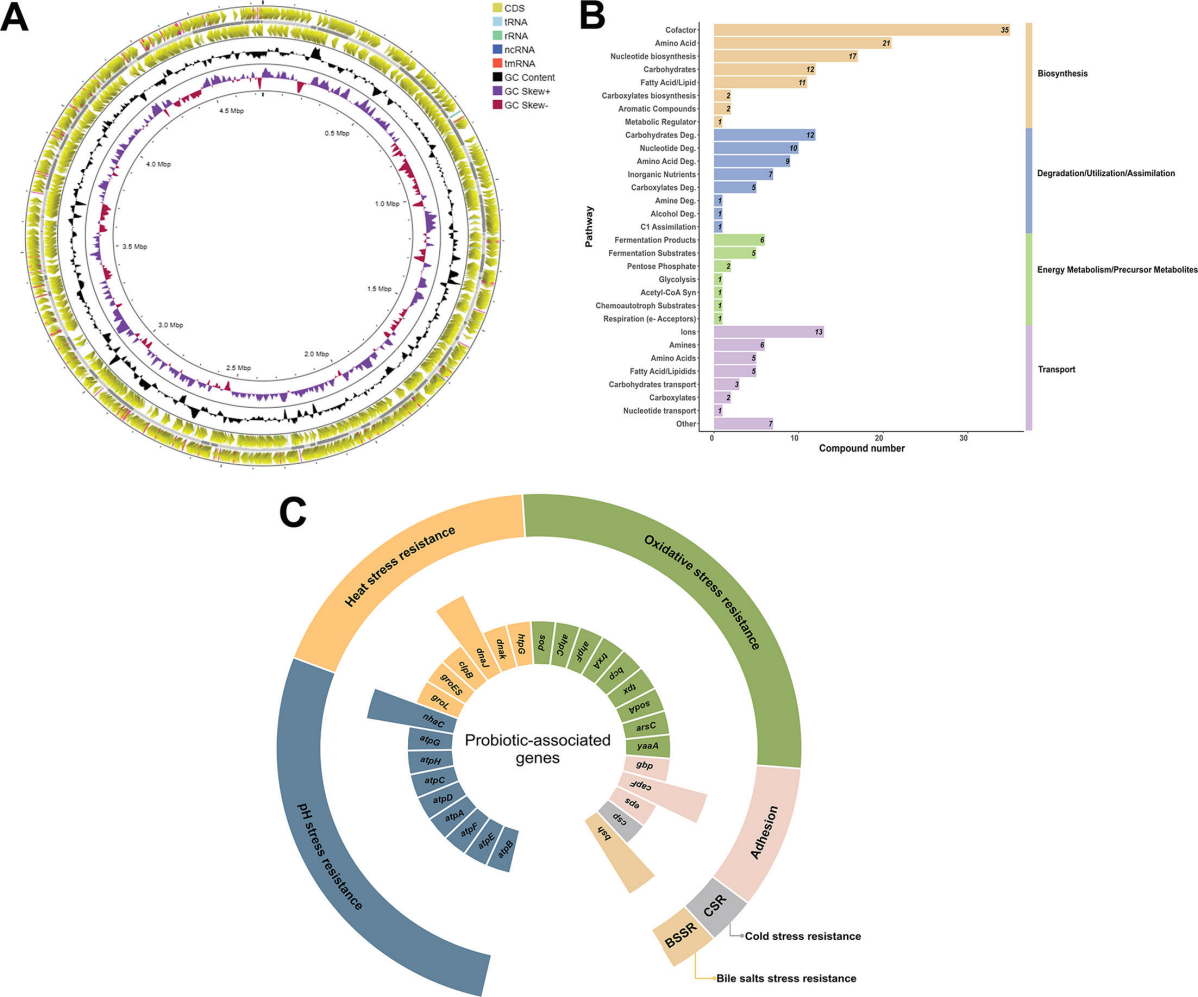
Genome overview and pathway distribution of *B. finegoldii* UO.H1052. (**A**) Draft genome visualization using the Proksee genome browser displaying CDSs, GC ratio, and GC skew. (**B**) Distribution of metabolic pathways with compound counts grouped into biosynthesis, degradation/utilization/assimilation, energy metabolism, and transport. (**C**) Distribution of genes associated with probiotic functions in *B. finegoldii* UO.H1052, including adhesion, pH, cold, heat, oxidative, and bile salt stress resistance traits, contributing to gut health. The outer track represents the main categories, whereas the inner track displays the detected genes. Bar length indicates gene count, with longer bars representing two genes and shorter bars representing one gene.

The whole genome sequence of *B. finegoldii* UO.H1052 (GenBank: JAQPYU000000000.1) is a single circular chromosome of 4,732,446 bp, with a 42.38% GC ratio. Genome annotation using PGAP yielded 3,943 genes, of which 3,758 were protein-coding genes (CDSs), 66 were RNA (rRNA, tRNA, and ncRNA) genes, and 119 were pseudogenes (Fig. 1A). Genome analysis using BioCyc, a comprehensive resource for biological pathways and genomes, identified 211 pathways in *B. finegoldii* UO.H1052. The distribution of these pathways across key biological processes, including biosynthesis, degradation-utilization-assimilation, energy metabolism/precursor metabolites, and transport, with compound counts provided for each pathway, is shown in Fig. 1B.

#### Horizontal gene transfer elements and metabolite biosynthetic clusters

Horizontal gene transfer, also known as lateral gene transfer, plays a key role in bacterial evolution. Prophage analysis using PHASTEST identified two distinct regions in the *B. finegoldii* UO. H1052 genome. The first region, classified as intact, comprised 40.3 kb, whereas the second region, an incomplete region, was 15.8 kb. These regions collectively contain 38 phage-related genes, none of which are associated with antimicrobial resistance or virulence factors (Fig. S1A). No CRISPR loci or associated *cas* genes were identified in the *B. finegoldii* UO.H1052 genome. The genomic islands (GIs) were predicted within the genome, and the analysis revealed 25 distinct genomic islands. These islands collectively contained 1,222 genes, with a significant proportion annotated as hypothetical proteins and others involved in diverse biological processes. Notably, no genes related to AMR or virulence were identified (Fig. S1B). To further determine antibiotic susceptibility, we measured the minimal inhibitory concentrations (MICs) of clinically relevant antibiotics *in vitro*. These antibiotics were selected based on their diverse mechanisms of action and to evaluate intrinsic and acquired resistance patterns. *B. finegoldii* UO.H1052 was susceptible to erythromycin (MIC = 1.2 µg/mL), tetracycline (MIC = 0.6 µg/mL), and ciprofloxacin (MIC = 6.2 µg/mL). In contrast, this strain showed intrinsic resistance to vancomycin (MIC > 150 µg/mL), gentamicin (MIC > 100 µg/mL), chloramphenicol (MIC > 50 µg/mL), and ampicillin (MIC > 50 µg/mL). In addition, antiSMASH analysis of the *B. finegoldii* UO.H1052 genome revealed the presence of three distinct biosynthetic gene clusters: a terpene precursor cluster, an RRE-element containing cluster, and an arylpolyene cluster. These findings indicate the strain’s genetic potential to synthesize diverse secondary metabolites, including terpenoid compounds, signaling peptides (mediated by the RRE element), and arylpolyenes, which may confer antioxidant or protective advantages.

#### Probiotic-associated genes

Genomic analysis of *B. finegoldii* UO.H1052 identified genes associated with probiotic functions, including stress resistance, adhesion, and bile salt tolerance. This strain harbors genes for pH stress resistance, such as the *atp* operon (*atpA-H*), encoding F0F1 ATP synthase subunits, which are crucial for energy homeostasis in acidic environments (28). Additionally, *nhaC* encodes a Na+/H+ antiporter for ion homeostasis and enhances acid tolerance. Heat stress resistance is enhanced by molecular chaperones (*groL, groES, clpB, dnaJ, dnaK,* and *htpG*) that facilitate protein refolding and prevent thermal denaturation (28). The oxidative stress resistance genes identified included *sod* and *sodA* (superoxide dismutases), *ahpC* and *ahpF* (alkyl hydroperoxide reductases), as well as *trxA, bcp,* and *tpx* (thioredoxin and peroxidase-related enzymes), which protect bacterial cells against oxidative damage (28) (Fig. 1C). Adhesion to host cells is crucial for bacterial colonization. The UO.H1052 genome encodes *gbp* (glycan-binding protein), *capF* and *eps* (capsular/exopolysaccharide biosynthesis), and *ompA* (outer membrane protein A), all of which are involved in host interaction and biofilm formation (28). Furthermore, tolerance to temperature alterations is highlighted by the presence of cold-shock domain-containing proteins (*csp*) (28). The genome also harbors choloylglycine hydrolase (*bsh*) genes, which are involved in bile salt resistance, a key trait for intestinal survival (28) (Fig. 1C).

### Metabolic profiling

**Fig 2:**
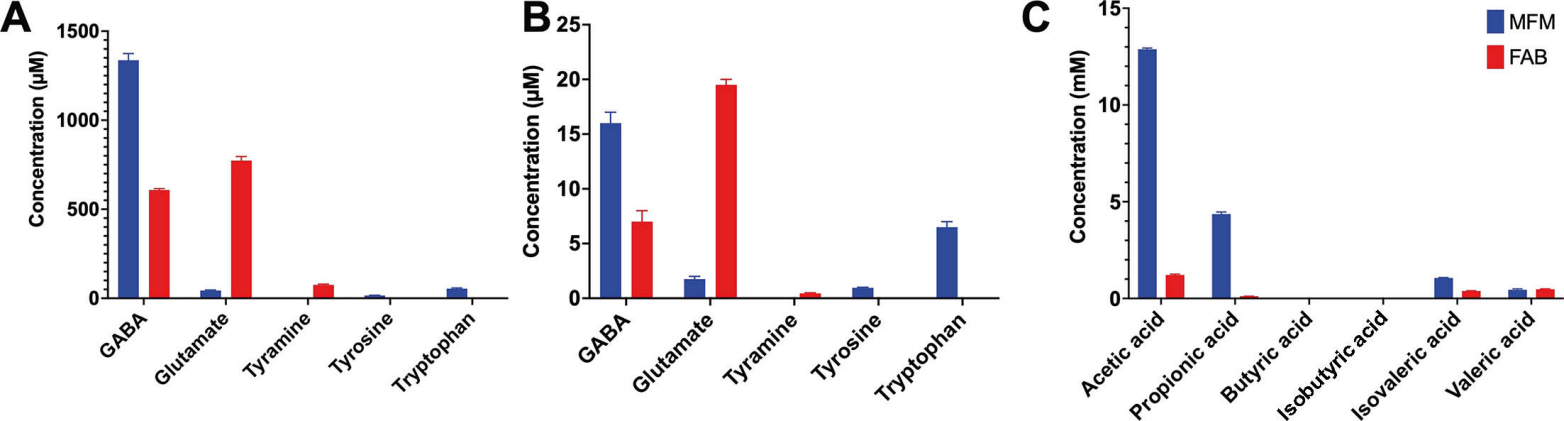
The metabolomic profiles of *B. finegoldii* UO.H1052-derived postbiotics are culture media-dependent. Metabolomic profiling of CFS and EVs generated by *B. finegoldii* UO.H1052 cultured in FAB (red) and Macfarlane (MFM; blue) media was performed using targeted LC-MS metabolomics (nano-flow LC–MS/MS). Identification and concentration (µM) of GABA, glutamate, tyrosine, tryptophan, and tyramine in CFS and EVs were determined based on retention time, *m/z* values, standard curves, and peak intensities of pure standard compounds, with a mass tolerance of 5 ppm and confirmed with MS1 and MS2. (**A**) Neuroactive metabolite concentrations in the CFS. (**B**) Neuroactive metabolites detected in EVs. (**C**) GC was used to determine the SCFA composition in CFS, and quantification was done based on a 10 mM SCFA mixture.

Targeted metabolomic profiling revealed that the production of neuroactive metabolites by *B. finegoldii* UO.H1052 was medium specific (Fig. 2). GABA was the most abundant metabolite detected in CFS, with concentrations reaching 1,337.0 ± 37.0 µM and 608.0 ± 8.0 µM in MFM and FAB, respectively. GABA levels in EVs were markedly lower, with 16.0 ± 1.0 µM and 7.0 ± 1.0 µM detected in MFM- and FAB-grown cultures, respectively. Glutamate (10 mM) was supplemented in the media and was extensively consumed, with FAB CFS retaining 773.0 ± 23.0 µM, whereas MFM CFS exhibited significantly lower levels (43.5 ± 3.5 µM). The inverse correlation between glutamate depletion and high GABA content in MFM CFS, along with its lower levels in FAB CFS, strongly indicated active glutamate decarboxylation to GABA. Glutamate was detected in EVs isolated from *B. finegoldii* grown in FAB and MFM at concentrations of 19.5 ± 0.5 µM and 1.75 ± 0.25 µM, respectively. Tyramine was exclusively detected in FAB-derived CFS (75.0 ± 5.0 µM) and EVs (0.45 ± 0.05 µM), while tyrosine and tryptophan were selectively produced in MFM-derived CFS and EVs. Tyrosine was detected at a concentration of 16.0 ± 1.0 µM in MFM CFS and 0.95 ± 0.05 in EVs, whereas tryptophan reached 54.0 ± 4.0 µM and 6.5 ± 0.5 µM in MFM CFS and EVs, respectively.

Similarly, SCFA quantification in the CFS produced by *B. finegoldii* UO.H1052 revealed a medium-dependent fermentation profile (Fig. 2C). When cultured in MFM, the strain produced significantly high levels of acetic acid (12.88 ± 0.06 mM), propionic acid (4.36 ± 0.11 mM), and butyric acid (3.04 ± 0.10 µM). Comparatively, cultures grown in the FAB medium produced markedly lower concentrations of acetic acid (1.21 ± 0.05 mM), propionic acid (120 ± 0 µM), and butyric acid (20 ± 0 µM). Similarly, isobutyric acid (0.69 vs 0.06 mM), isovaleric acid (1.46 ± 0.02 mM vs 0.39 ± 0.02 mM), and valeric acid (1.03 ± 0.03 mM in both media) followed this trend, with MFM consistently supporting higher SCFA production.

### Survival under gastrointestinal conditions

**Fig 3:**
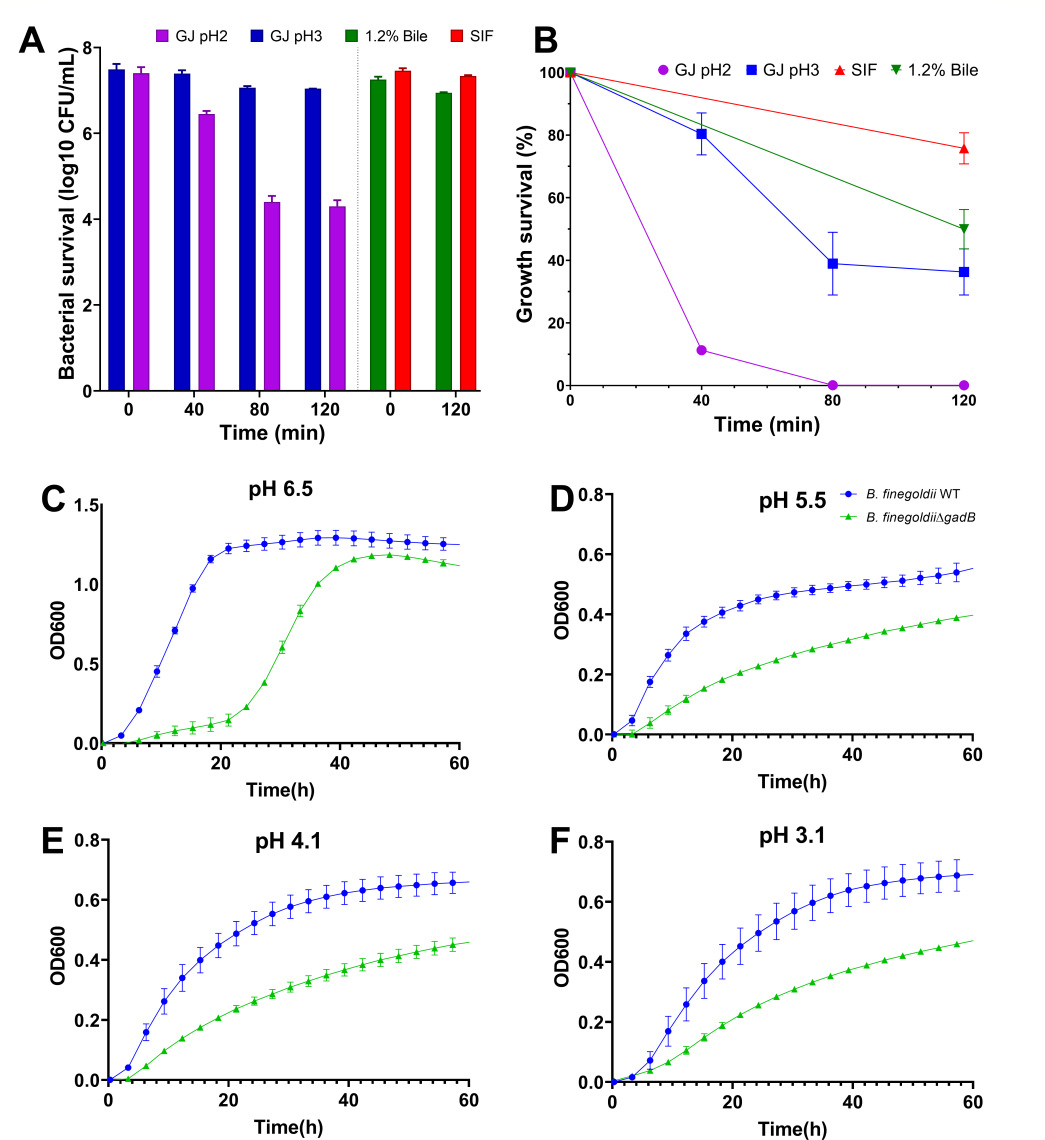
Acid and bile tolerance and GABA-linked acid resistance mechanisms in *B. finegoldii* UO.H1052. (**A**) Survival dynamics of *B. finegoldii* UO.H1052 upon exposure to simulated gastrointestinal conditions, including gastric juice (GJ) at pH 2 and 3, 1.2% bile, and SIF. Bacterial viability was quantified at defined time points and expressed as Log₁₀ CFU/mL. (**B**) Percentage of survival under each condition relative to controls, highlighting tolerance variability across stressors. (**C–F**) Growth kinetics of *B. finegoldii* UO.H1052 wild-type (blue circle) and the Δ*gadB* mutant (green triangle) in minimal media supplemented with xylose as the sole carbon source, suggesting GABA as an acid resistance mechanism.

The viability of *B. finegoldii* UO.H1052 was evaluated under simulated gastric and intestinal conditions. In simulated gastric juice (SGJ) at pH 2, viability significantly declined from 7.40 ± 0.10 log CFU/mL at baseline to 6.45 ± 0.05 log CFU/mL (approximately 12.2% survival) after 40-min exposure. Viability further decreased sharply to 4.40 ± 0.10 log CFU/mL (0.12% survival) at 80 min and to 4.30 ± 0.10 log CFU/mL (0.10% survival) after 120 min (Fig. 3A and B). Conversely, exposure to simulated gastric juice at pH 3 resulted in minimal reductions from 7.49 ± 0.09 log CFU/mL at baseline to 7.39 ± 0.05 log CFU/mL (88.7% survival) at 40 min and then declined slightly further to approximately 7.07 ± 0.03 log CFU/mL (around 35% survival) at both 80 and 120 min (Fig. 3A and B). In simulated intestinal fluid (SIF), the bacterial counts remained stable, slightly declining from 7.46 ± 0.04 log CFU/mL at baseline to 7.34 ± 0.01 log CFU/mL (97% survival) after 120 min. Exposure to 1.2% bile showed a modest reduction from an initial count of 7.25 ± 0.05 log CFU/mL to 6.95 ± 0.01 log CFU/mL, reflecting approximately 50% survival after 120 min of incubation (Fig. 3A and B).

The effect of acid stress on the growth and survival of *B. finegoldii* UO.H1052 (WT) was investigated by comparing wild-type bacteria with a recently developed Δ*gadB* null mutant (26) (Fig. 3C). The WT and Δ*gadB* mutant strains of *B. finegoldii* UO.H1052 exhibited robust growth at pH 6.5, with the mutant growing at a slightly slower rate. However, the Δ*gadB* mutant showed a significant decrease in the growth rate at pH 5.5, 4.1, and 3.1. This indicates that GABA plays a critical role in enhancing acid resistance.

### Adhesion of *Bacteroides finegoldii* UO.H1052 to Caco-2/HT29-MTX cells and cytotoxicity assays

**Fig 4:**
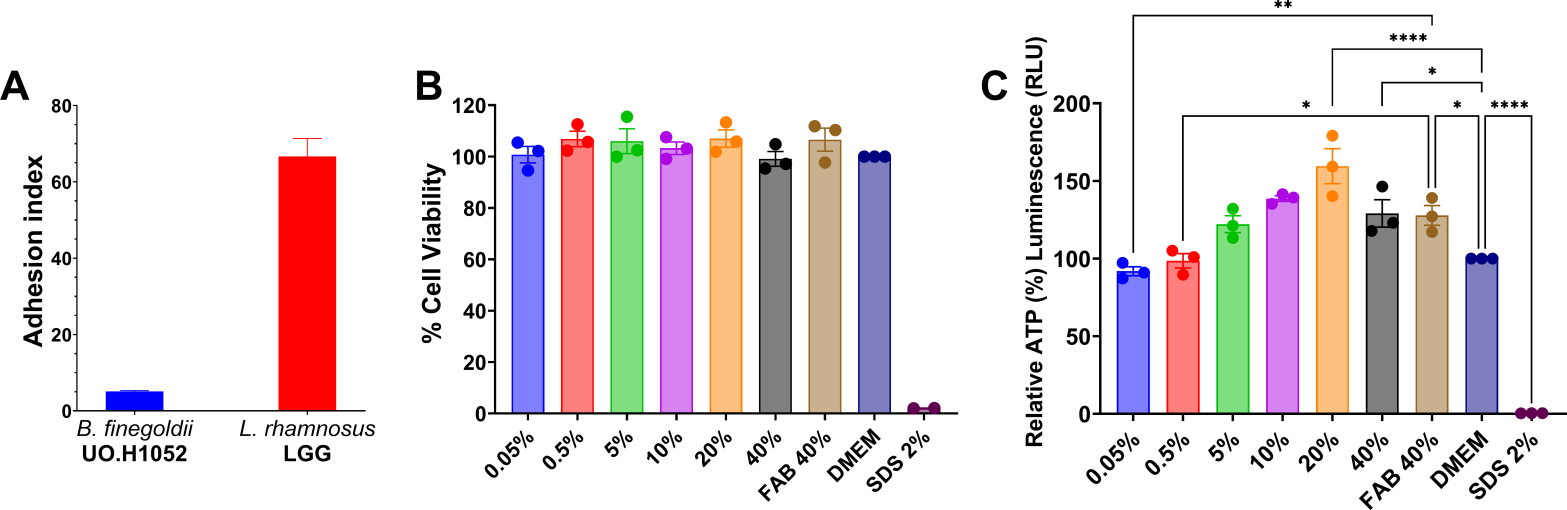
Adhesion, cytotoxicity, and metabolic activity profiling of *B. finegoldii* UO.H1052 in an intestinal epithelial cell model (Caco-2/HT29-MTX). (**A**) The adhesion capacity of *B. finegoldii* UO.H1052 to the Caco-2/HT29-MTX co-culture model was quantified as the adhesion index (mean number of bacteria adhered per host cell), with LGG included as a probiotic benchmark. (**B**) Cytotoxicity was assessed via LDH release (CytoTox 96 Assay) in a Caco-2/HT29 (9:1) co-culture model after 24-h treatment with CFS (0.5%, 5%, 10%, 20%, and 40%). Dulbecco’s modified Eagle medium was used as the negative control, SDS was used as the cytotoxic control, and FAB was used as the vehicle control. (**C**) Cellular ATP levels were measured using ATP-based viability assay (CellTiter-Glo) after CFS exposure to evaluate epithelial metabolic activity and barrier integrity. The results showed a dose-dependent increase in ATP levels, which peaked at 20% CFS.

Quantitative adhesion analysis revealed that approximately 1.7 × 10⁵ CFU of *B. finegoldii* UO.H1052 adhered to the Caco-2/HT29 monolayer, an *in vitro* model for human intestinal epithelium commonly used to assess gut barrier function and host-microbe interactions, when seeded with 2.0 × 10^8^ CFU/mL. Adhesion index was calculated to determine the number of bacteria adhered to each cell (Fig. 4A). Notably, increasing the seeding density to 1.0 × 10^9^ CFU/mL resulted in a marked rise in adhesion, with 2.53 × 10⁶ CFU successfully attaching to the simulated epithelial monolayer. These findings indicated the adhesion capacity of *B. finegoldii* UO.H1052, highlighting its affinity for intestinal epithelial cells under *in vitro* conditions. *Lacticaseibacillus rhamnosus* GG (LGG), used as a probiotic reference control, exhibited a higher adhesion index.

The cytotoxicity of the *B. finegoldii* UO.H1052 CFS was assessed on Caco-2/HT29 model using ATP and LDH assays (Fig. 4B and C). ATP assay results demonstrated no cytotoxicity; instead, ATP levels increased in a concentration-dependent manner, indicating enhanced metabolic activity, particularly at higher CFS concentrations (158.78% ± 14.47% at 20% CFS) (Fig. 4B). The LDH release assay corroborated these findings, showing no substantial cytotoxic effects, as LDH release remained near the control levels (near 100%) across all tested concentrations (Fig. 4C).

### *Bacteroides finegoldii* UO.H1052 enhances barrier integrity, induces serotonin secretion *in vitro*, and exhibits immuno-stimulatory properties

**Fig 5:**
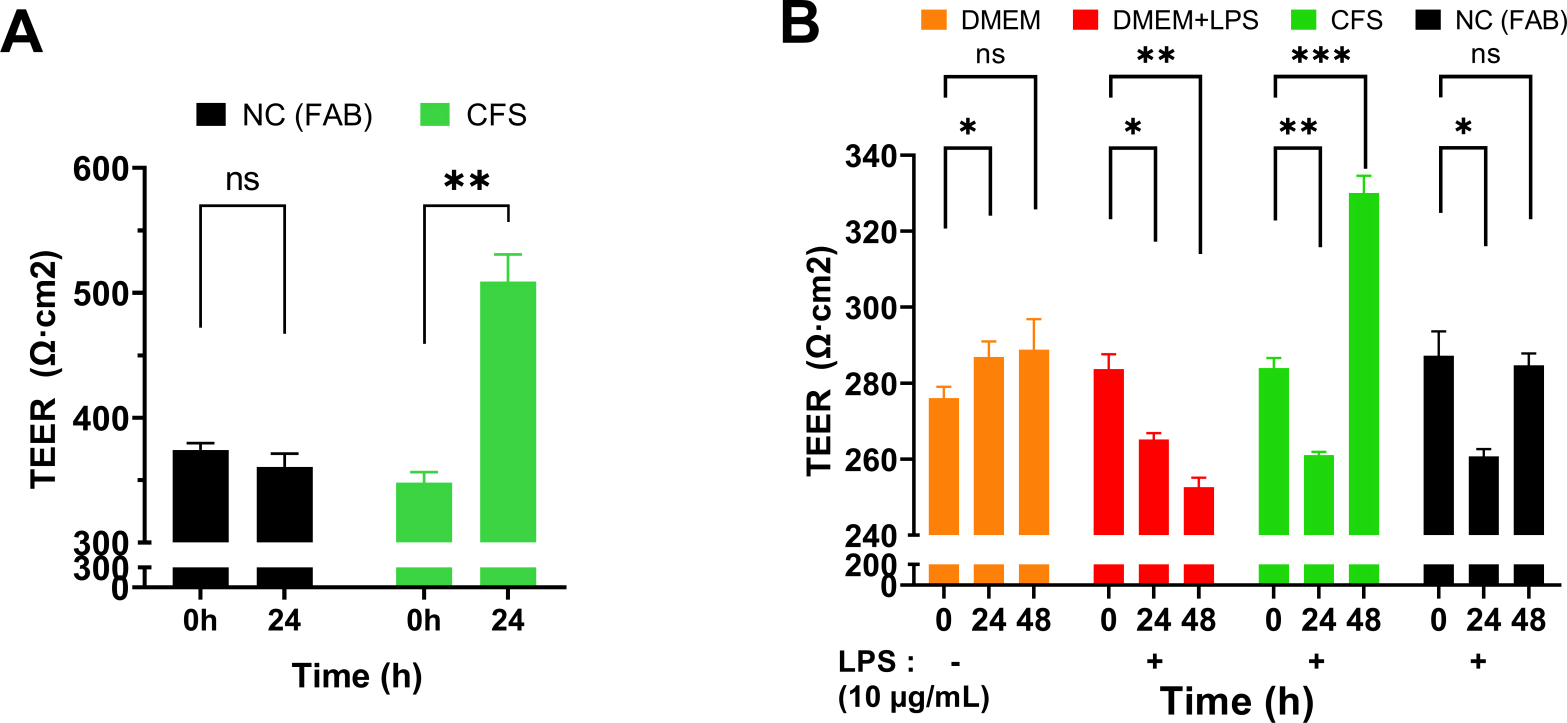
*B. finegoldii* UO.H1052 CFS enhances intestinal barrier integrity. (**A**) Caco-2 monolayers grown on Transwell inserts for 21 days were treated with *B. finegoldii* CFS (20%) for 24 h, resulting in a significant increase in TEER compared to the FAB media control, indicating improved barrier function. (**B**) CFS restored TEER in an LPS-induced leaky gut model. Caco-2/HT29 co-cultures grown for 21 days were exposed to LPS (10 µg/mL) for 24 h, which led to a significant reduction in TEER. Control monolayers (DMEM only) remained stable, while subsequent CFS treatment significantly increased TEER even higher than the LPS control, mitigating LPS-induced leaky gut conditions.

To evaluate the effect of *B. finegoldii* UO.H1052 on epithelial barrier integrity, transepithelial electrical resistance (TEER) was measured in Caco-2/HT29-MTX co-cultures at baseline (0 h) and after 24 h of CFS treatment (Fig. 5A). At baseline, TEER values were comparable between the negative control (NC; 374.25 ± 9.39 Ω·cm²) and the CFS-treated group (348.00 ± 14.74 Ω·cm²). However, after 24 h, cultures treated with *B. finegoldii* CFS exhibited a significant increase in TEER (509.00 ± 37.74 Ω·cm²), while the NC group showed a slight and non-significant decrease (360.72 ± 18.09 Ω·cm²). These results indicate that CFS enhanced epithelial barrier integrity under the tested conditions. The protective effect of CFS against LPS-induced barrier disruption was assessed in a Caco-2/HT29-MTX co-culture (Fig. 5B). Following LPS exposure (24 h), all experimental groups, except the Dulbecco’s modified Eagle medium (DMEM) negative control, showed decreased TEER values (Fig. 5B), reflecting compromised barrier integrity. However, after an additional 24 h (total 48 h), cultures treated with CFS showed a significant recovery, with TEER increasing to 330.03 ± 3.71 Ω·cm², surpassing the initial baseline and all other groups. In contrast, cultures exposed only to DMEM + LPS continued to deteriorate, reaching a minimal TEER of 252.62 ± 2.03 Ω·cm². The untreated control medium and DMEM groups maintained relatively stable TEER throughout the experiment. These results demonstrated the ability of *B. finegoldii* CFS to strengthen and recover epithelial barrier integrity following LPS-induced disruption.

**Fig 6:**
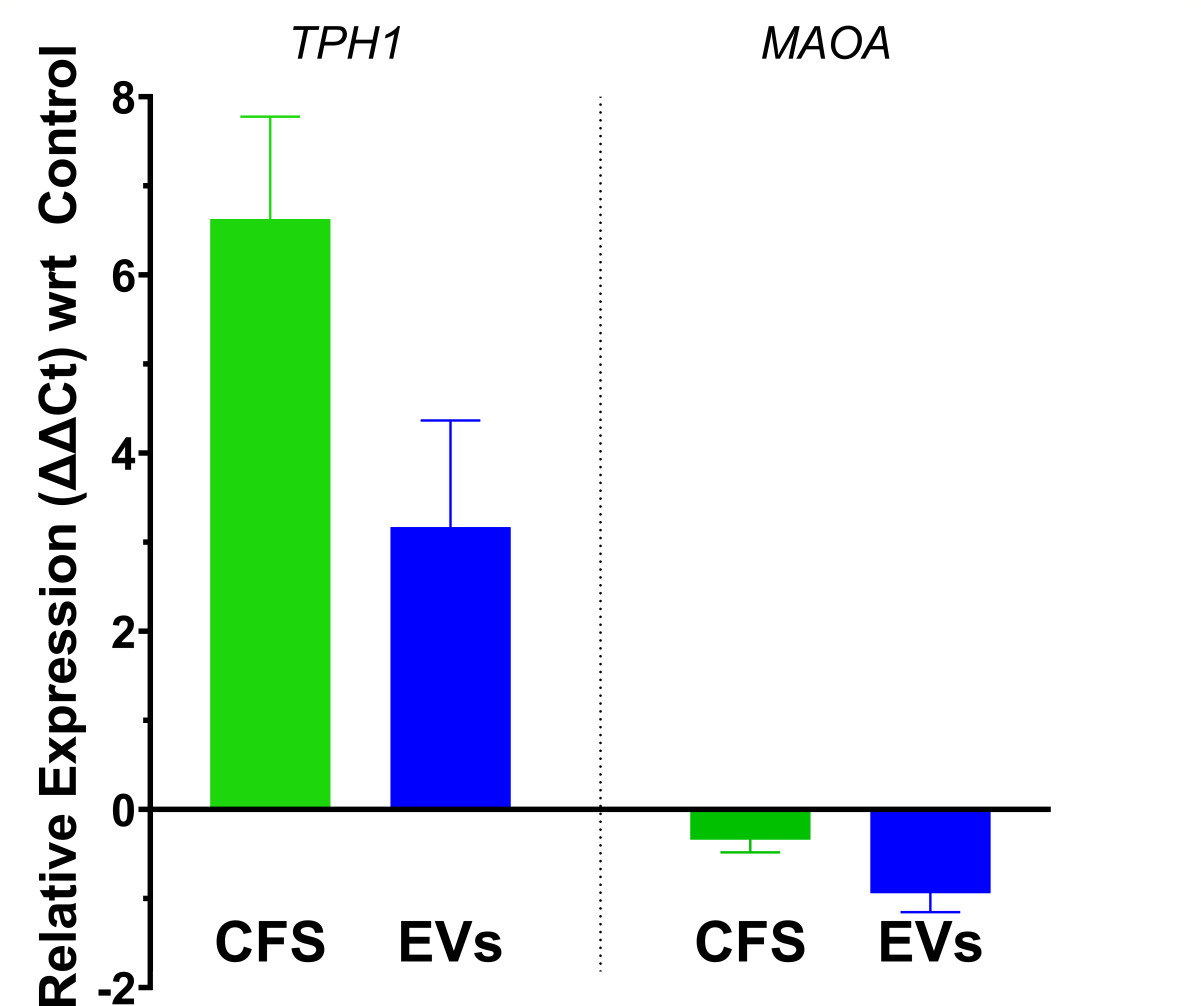
*B. finegoldii* UO.H1052-derived postbiotics modulate serotonin metabolic pathways in enteroendocrine cells. RT-qPCR analysis of *Tph1* and *Maoa* gene expression in RIN14B enteroendocrine cells after 6-h treatment with CFS and EVs. Gene expression was calculated relative to the FAB medium control. PBS served as a vehicle/background control using the ΔΔCt method and was normalized to the β-actin housekeeping gene. Both CFS and EVs induced differential transcriptional regulation of serotonergic markers.

To assess the effect of *B. finegoldii* UO.H1052-derived postbiotics on serotonin metabolism, we quantified the transcriptional responses of the key serotonergic genes, *Tph1* and *Maoa* (monoamine oxidase), in RIN14B enteroendocrine cells following treatment with CFS and EVs. The EV concentration, as determined by nanoparticle tracking analysis, was 3.07 × 10¹⁰ particles/mL and subsequently used at various dilutions for *in vitro* functional assays (Fig. S3). As shown in Fig. 6, *Tph1* expression was markedly upregulated in both treatment groups, with CFS eliciting a robust 6.63 ± 0.94-fold induction, compared to a 3.17 ± 0.98-fold increase by EVs. In contrast, *Maoa* expression exhibited a slight downregulation in response to both treatments (EVs: −0.94 ± 0.15-fold; CFS: −0.34 ± 0.10-fold), though these changes did not reach statistical significance (*P* = 0.096). These findings indicate that these postbiotics selectively enhanced serotonin biosynthesis without substantially affecting its catabolic degradation, with CFS demonstrating a more potent effect on *Tph1* activation.

**Fig 7:**
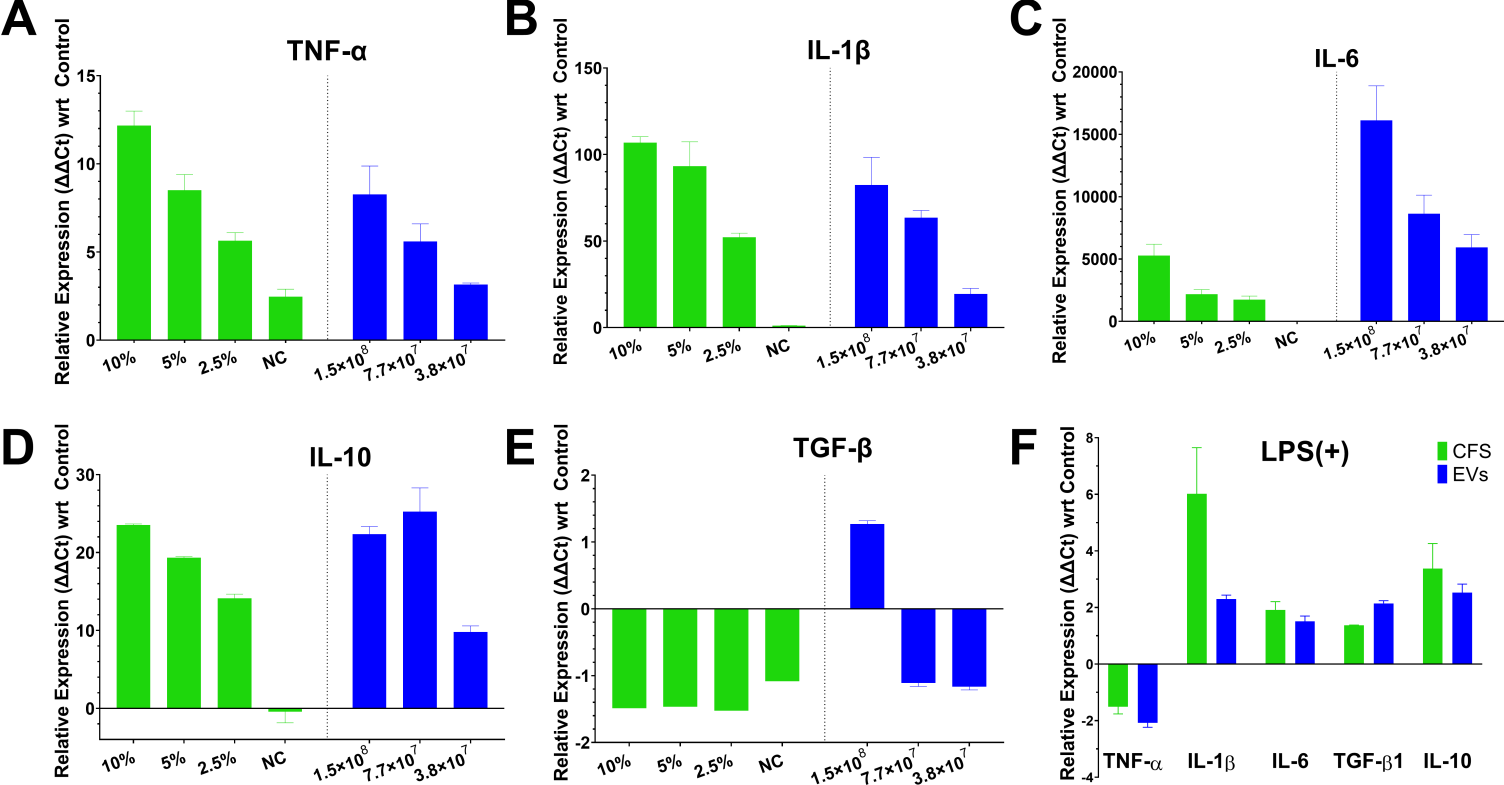
*B. finegoldii* UO.H1052-derived postbiotics modulate cytokine expression in RAW264.7 macrophages under basal and inflammatory conditions. RT-qPCR analysis of inflammatory and anti-inflammatory gene expression in RAW264.7 macrophages following treatment with CFS and EVs. Gene expression was calculated relative to untreated control samples using the ΔΔCt method and normalized to β-actin. (**A-E**) In the absence of LPS, both CFS and EVs significantly upregulated inflammatory (*Tnf*-α, *Il-1β*, and *Il-6*) and anti-inflammatory (*Il-10*) cytokines, indicating immunostimulatory potential and a role in maintaining immune homeostasis. (**F**) In LPS-challenged macrophages, 24-h treatment with CFS or EVs markedly suppressed pro-inflammatory *Tnf-α* expression and enhanced anti-inflammatory *Il-10* and *Tgf-β* expression, reflecting immunomodulatory activity.

Gene expression profiling revealed distinct immunomodulatory effects of *B. finegoldii* UO.H1052-derived postbiotics on inflammatory cytokine regulation in RAW 264.7 macrophages (Fig. 7). In unstimulated conditions (absence of LPS), both CFS and EVs elicited potent, dose-dependent immunostimulatory responses (Fig. 7A through E). The extent of cytokine upregulation varied substantially, with *Il-6* demonstrating an exceptionally high induction (10,000- to 20,000-fold), followed by *Il-1β* (~100-fold), and *Tnf-α* (~20-fold) upon treatment with 10% CFS and EVs at 1.54E+08 particles/mL. Notably, cytokine induction declined significantly in a dose-dependent manner. The anti-inflammatory cytokine *Il-10* was also markedly upregulated (~20-fold), whereas *Tgf-β1* exhibited only minor changes in its expression. Across all cytokines under unstimulated conditions, CFS consistently evoked stronger gene expression responses than EVs, except for *Il-6*, for which EVs showed stronger upregulation. Under LPS-stimulated inflammatory conditions (5 µg/mL), both CFS and EVs significantly suppressed *Tnf-α* expression (Fig. 7F). EVs demonstrated a slightly higher inhibitory capacity (−2.08 ± 0.16-fold) relative to CFS (−1.51 ± 0.36-fold). Moreover, CFS markedly enhanced *Il-1β* expression (6.01 ± 2.31-fold), while EVs induced a more moderate increase (2.30 ± 0.14-fold). Modest elevations in *Il-6* levels were observed following both treatments (1.91 ± 0.42-fold for CFS; 1.51 ± 0.19 for EVs). Additionally, both postbiotics upregulated anti-inflammatory mediators *Tgf-β1* (2.14 ± 0.10-fold vs 1.37 ± 0.01 for CFS) and *Il-10* levels (2.52 ± 0.30-fold vs 3.37 ± 1.25 for CFS).

## DISCUSSION

The human gastrointestinal tract hosts a dense and diverse microbial ecosystem, with Bacteroidota constituting a dominant and functionally vital phylum (6). Emerging evidence has revealed significant inverse associations between gut *Bacteroides* abundance and depression (15), along with positive correlations with increased gray matter in brain regions involved in mood regulation, including the cerebellum, hippocampus, and the frontal cortex (29). In preclinical models, high-fat diets were shown to reduce *Bacteroides* abundance and cortical GABA levels, concomitant with depressive-like behaviors (30), linking *Bacteroides*, GABA production, and the gut-brain axis to mood regulation. Recent studies have increasingly recognized Bacteroidota species as compelling candidates for NGPs (4, 5). Despite their therapeutic potential, relatively few *Bacteroides* strains have been evaluated as probiotics. In this context, we investigated *B. finegoldii* UO.H1052 as a novel psychobiotic candidate with potential to modulate gut-brain axis signaling.

Whole-genome sequencing confirmed the absence of known virulence factors and transferable antibiotic resistance genes, thus supporting a favorable safety profile. *In vitro* MIC testing revealed susceptibility to clinically relevant antibiotics, including erythromycin, tetracycline, and ciprofloxacin, while resistance to vancomycin, gentamicin, chloramphenicol, and ampicillin was observed, an expected profile for Bacteroidota species due to their intrinsic, chromosomally encoded resistance mechanisms (31). Although *Bacteroides* species hold potential as probiotics, their use is often constrained by the opportunistic pathogenicity observed in some strains under specific conditions, necessitating rigorous safety assessment. To date, only a limited number of *Bacteroides* strains, namely *B. fragilis* ZY312, *B. uniformis* CECT7771, *Bacteroides xylanisolvens* DSM23964, *B. dorei*, and *B. ovatus* ELH-B2, have been evaluated for safety and reported as promising probiotic candidates with potential therapeutic effects on gut-related disorders (32–34). In this study, CFS produced by *B. finegoldii* UO.H1052 showed no cytotoxicity in Caco-2 cells. These data suggest that *B. finegoldii* UO.H1052 CFS does not negatively affect epithelial cell viability and may enhance cellular metabolic functions.

Metabolomic profiling revealed that *B. finegoldii* UO.H1052 produces neuroactive compounds such as GABA, tyramine, tryptophan, and tyrosine, with GABA being the most abundant. These findings further support the prominent role of *Bacteroides* and *Parabacteroides* species in shaping intestinal GABA content (15, 26). GABA, the primary inhibitory neurotransmitter in the mammalian brain, exerts pleiotropic effects on immune modulation (26) and mood disorders (15). Notably, *B. ovatus* ATCC 8483, a closely related species, has been shown to increase intestinal GABA levels in murine models (35). Recently, both GABA and tyramine, along with dopamine and kynurenine, have been identified as plasma biomarkers capable of distinguishing between individuals with and without depression (36). Although the ability of gut-derived neuroactive compounds to cross the blood-brain barrier remains debated, emerging evidence suggests that they may modulate brain function via gut-brain signaling, including vagal nerve activation or gut epithelial signaling, or limited BBB penetration (37). GABA has also recently been suggested as a promising postbiotic for the management of neurological and psychological disorders (38). Intriguingly, neuroactive molecules such as GABA, tyramine, tryptophan, and tyrosine were also identified within EVs at low micromolar concentrations, suggesting a vesicle-mediated route for delivery to distal host tissues, including the brain (27).

Furthermore, supporting its psychobiotic capacity, *B. finegoldii* UO.H1052 produced substantial levels of SCFAs under fiber-rich conditions, primarily acetate. SCFAs are key mediators of neuro-immunoendocrine communication and contribute to the maintenance of BBB integrity, attenuation of neuroinflammation, and modulation of brain function (39). Although acetate is widely produced by diverse microbial taxa (40), the production of propionate and butyrate tends to be species-specific. 

Beyond its safety features, *B. finegoldii* UO.H1052 exhibits several functional traits that are characteristic of promising probiotics. The strain maintained a high viability under simulated gastrointestinal conditions, including survival under high bile salt concentrations and low pH stress. GABA production also contributed to acid resistance, as confirmed by growth analyses of *B. finegoldii* and its isogenic Δ*gadB* mutant (26). In addition, *B. finegoldii* considerably adheres to Caco-2/HT29 epithelial cells, a critical feature for transient colonization and interaction with host tissues. In contrast, *B. xylanisolvens* DSM 23964 was previously reported to lack adhesion to Caco-2 cells (41). Additionally, our findings demonstrated that CFS significantly enhances epithelial barrier integrity. Notably, CFS treatment restored compromised epithelial function following LPS challenge. These results align with recent reports showing that *B. fragilis* ZY-312 can repair intestinal barrier damage induced by radiotherapy and ulcerative colitis (10, 42).

Another key functional attribute of *B. finegoldii* postbiotics (CFS and EVs) is their ability to significantly upregulate the expression of *Tph1*, a rate-limiting enzyme in serotonin biosynthesis. Previous studies have demonstrated that the gut microbiota promotes serotonin biosynthesis in enterochromaffin cells, which in turn supply serotonin to the intestinal mucosa, lumen, and circulating platelets (43). Gut-derived serotonin plays multifaceted roles in host physiology, including regulation of gastrointestinal motility, immune modulation, and cardiovascular function (44, 45). Several neurotransmitters produced in the gut, such as GABA, serotonin, dopamine, tyramine, and kynurenine, have been implicated in mood regulation and the pathophysiology of depressive disorders (36). GABA is predominantly produced by members of the phylum Bacteroidota, whereas serotonin is synthesized by enterochromaffin cells (46, 47), both of which contribute to gut-brain communication, immune homeostasis, and intestinal motility. Interestingly, some *Bacteroides* strains, such as *B. fragilis* ATCC 25285 and *B. uniformis* ATCC 8492, have been associated as biomarkers in major depressive disorder, potentially via modulation of tryptophan metabolism and gut-derived neurotransmitters (48). In contrast, other strains, including *B. vulgatus* and *B. uniformis* CECT 7771, have been shown to alleviate or negatively associate with depressive symptoms through gut-brain axis interactions (49). These opposing behavioral outcomes within the *Bacteroides* genus underscore the complexity of microbial contributions to host neurobiology, highlighting the need for further mechanistic elucidation. GABA-producing *Bifidobacterium dentium* has also been shown to influence serotonergic signaling in gnotobiotic mice by modulating 5-HT receptor expression in both the gut and the brain (50).

The postbiotics (CFS and EVs) induced both upregulation of anti-inflammatory *Il-10* and pro-inflammatory cytokines (*Tnf-α*, *Il-1β*, and *Il-6*), with a high *Il-10*/*Tnf-α* ratio. Given *Il-10*’s well-established role in suppressing antigen presentation via MHC class II downregulation (20), *B. finegoldii* postbiotics may contribute to immune homeostasis. These findings are consistent with previous reports demonstrating that EVs from *Bacteroides* species can mediate *Il-10*-driven immunoregulation. For example, *B. thetaiotaomicron* EVs have been shown to ameliorate colon inflammation via systemic *Il-10* induction (51, 52), whereas EVs from commensals promote *Il-10* production by regulatory T cells, contributing to mucosal immune tolerance (23, 53). The therapeutic relevance of these mechanisms is further supported by emerging proposals to develop *Bacteroides*-derived EVs as treatments for IBD (21) and their exploitation in mucosal vaccine delivery (54). Another notable observation was the strong induction of *Il-6* by *B. finegoldii* postbiotics, a cytokine known for its context-dependent role in mediating both acute immune defenses and chronic inflammation (55). Extreme *Il-6* elevation alongside substantial *Il-10* production has been suggested to have potent innate immune stimulation and compensatory anti-inflammatory responses, respectively (53). In LPS-challenged macrophages, the concurrent upregulation of *Tgf-β* and *Il-10*, along with the suppression of *Tnf-α*, further underscores the anti-inflammatory potential of *B. finegoldii* postbiotics, supporting their role in promoting immune homeostasis. In addition to *Bacteroides*, *Lactobacilli* have also been recognized for their immunostimulatory activity. For example, heat-treated *Levilactobacillus brevis* KU15159 significantly upregulated the production of pro-inflammatory cytokines (*Tnf-α*, *Il-1β*, and *Il-6*), inducible nitric oxide synthase, and nitric oxide (NO) in macrophages, mediated through the MAPK signaling pathway (56). Similarly, conditioned media from multispecies probiotics significantly enhanced macrophage phagocytosis, *Tnf-α* production, and NO release, indicating their strong immunostimulatory capacity (57).

In summary, our findings demonstrate that *B. finegoldii* UO.H1052 and its postbiotics exhibit a favorable safety profile, with no detectable cytotoxicity, and possess dual immunomodulatory and neuroactive capabilities. Their ability to enhance epithelial barrier integrity and induce serotonin production highlights the strain’s psychobiotic potential for gut-brain axis modulation. The nanoscale size, stability, and non-replicative nature of the derived EVs further support their utility as biotherapeutic agents. Future studies in animal models are warranted to evaluate the safety, psychobiotic potential, and translational relevance for the treatment of intestinal barrier dysfunction and neuroinflammatory and depressive disorders.

## MATERIALS AND METHODS

### Strains and culture conditions

*B. finegoldii* UO.H1052 was isolated from the stool of a healthy Canadian female (26) and cultured in FAB and/or supplemented with glutamate (10 mM) under anaerobic conditions (85% N_2_, 10% CO_2_, and 5% H_2_) until the exponential phase (48–78 h). Secondary cultures were inoculated into FAB or MFM, grown to the stationary phase, and used for further experimentation.

### Preparation of CFS, extraction of EVs, and characterization

CFS was obtained by centrifugation (7,500 × *g* for 30 min) and filtration (0.2 µm) and stored at −80°C. EVs were extracted by ultracentrifugation (45,000 × *g* for 1 h) of the CFS, washed, and resuspended in PBS. Size and concentration were determined using the ZetaView nanoparticle tracking system (Particle Metrix, Germany), and the data were analyzed using ZetaView software (version 8.02.28).

### Genome analysis for plasmids, virulence genes, and antibiotic resistance

Eighteen *Bacteroides* isolates, including *B. finegoldii*, were recently sequenced (58). These 18 genomes were investigated for antibiotic resistance genes using the Comprehensive Antibiotic Resistance Database (CARD) (59). Plasmids were predicted using the MOB suite and were further screened for antimicrobial resistance genes using AMRFinderPlus (version 3.11.26) (60, 61). Toxin-antitoxin systems were characterized using TADB (version 3.0) (62). Virulence factors were predicted using the Virulence Factor Database (VFDB) (63) and the ABRicate pipeline (https://github.com/tseemann/ABRicate) with the NCBI database (version 1.0.1).

### Genome characteristics of *Bacteroides finegoldii* UO.H1052

The UO.H1052 annotated genome was visualized using the Proksee server (https://proksee.ca/), and metabolic pathway analysis was performed using the Pathway Tools software (version 28) (64) utilizing the PGAP-annotated genome. Prophage regions were identified and annotated using PHASTEST (65), whereas the CRISPR loci (version 5.0.11) were employed for CRISPR system prediction (66). GIs were predicted, annotated, and visualized using IslandViewer version 4 (67). The pathway compounds and genes associated with probiotic traits were generated using R (version 4.3.1) (https://www.R-project.org/) within the RStudio 2024.12.1 Build 563 environment utilizing the ggplot2 package (version 3.5.1). Additionally, secondary metabolite biosynthetic gene clusters were predicted using antiSMASH (version 8.0.1) with default parameters (68).

### Metabolic profiling of CFS and EVs produced by *Bacteroides finegoldii *UO.H1052

#### Quantification of SCFAs using gas chromatography

SCFAs were quantified using gas chromatography with a flame ionization detector, as described previously (26). Briefly, 1 µL of CFS obtained from *B. finegoldii* UO.H1052 cultures grown in FAB or MFM was injected into the GC instrument. The temperature was set at 240°C for the injector and 280°C for the detector. The final oven temperature was increased at 2°C/min and maintained at 240°C for 5 min. The peaks of SCFAs were determined by comparing their retention times with standard references from Millipore Sigma (Oakville, ON, Canada) and quantified relative to a 0.5 mM 2-ethyl butyric acid internal standard. The SCFA concentration was expressed in millimolar.

#### Quantification of neuroactive metabolites using nLC-MS/MS

To analyze neuroactive metabolites, targeted metabolomics was conducted on CFS and EVs obtained from *B. finegoldii* UO.H1052 cultures grown in FAB and MFM. Nanoflow liquid chromatography-tandem mass spectrometry (nLC-MS/MS) was employed with commercial standards, including GABA, glutamate, tryptophan, 5-hydroxytryptophan, kynurenic acid, normelatonin, L-tyrosine, norepinephrine, dopamine, tyramine, and spermine (Sigma-Aldrich) for standard curve preparation (26). Raw spectral data were processed using the Xcalibur software to detect peaks, and quantification was achieved by calculating the peak areas against standard curves. Identification was confirmed based on the retention times and *m/z* values of precursor and fragmentation ions corresponding to the reference standards.

### Susceptibility of *Bacteroides finegoldii* to different antibiotic classes *in vitro*

Antibiotic susceptibility of *B. finegoldii* UO.H1052 was assessed against key antibiotics critical to human medicine, including penicillins (ampicillin), fluoroquinolones (ciprofloxacin), macrolides (erythromycin), glycopeptides (vancomycin), aminoglycosides (gentamicin), and tetracyclines (tetracycline). MICs were determined as the lowest antibiotic concentrations that completely inhibited bacterial growth, with a tested range of 0.125–100 µg/mL. *B. finegoldii* UO.H1052 was cultured in FAB medium under anaerobic conditions for 48 h. MIC assays were performed in 96-well plates using twofold serial dilutions of antibiotics in Mueller-Hinton broth supplemented with hemin and vitamin K (MHB-HK). Bacterial suspensions (10^5^ CFU/mL) in MHB-HK were prepared according to the Clinical and Laboratory Standards Institute guidelines. The positive controls contained bacterial inoculum without antibiotics, whereas the negative controls lacked the inoculum. The plates were incubated at 37°C in an anaerobic chamber, and optical density measurements were recorded every 20 min for 60 h using a Stratus plate reader (Cerillo, Charlottesville, VA, USA).

### Viability of *Bacteroides finegoldii *UO.H1052 after exposure to gastric acid, intestinal juice, and bile salts

The tolerance of *B. finegoldii* to gastrointestinal conditions was assessed by culturing it in FAB for 72 h, followed by washing and resuspension in PBS to 10^8^–10^9^ CFU/mL. For acid tolerance, bacterial suspensions were exposed to SGJ (2 g/L NaCl and 3.2 g/L pepsin, pH 2 and 3) for 0, 40, 80, and 120 min. For intestinal tolerance, suspensions were exposed to simulated intestinal juice (6.8 g/L KH_2_PO_4_ adjusted to pH 7.0, 10 g/L pancreatin, and 0.5 g/L bile salts) for 4 h. Bile salt tolerance was evaluated using 1.2% Oxgal in FAB incubated for 4 h. Samples were collected at 0 h and at subsequent time points from each test, serially diluted, plated on FAB agar (FAA), and incubated under anaerobic conditions at 37°C. The survival capacity (%) was calculated as 100 × (log CFU/mL)_TF_/(log CFU/mL)_T0_, where T0 and TF represent colony-forming units per milliliter at the start (0 h) and specific time points, respectively. Control samples were processed in parallel without exposure. In addition, the effect of acid stress on the growth and survival of *B. finegoldii* UO.H1052 was investigated by growing the strain and a recently constructed genetically engineered mutant (Δ*gadB*) (26) in minimal medium with xylose as the sole carbon source (0.5%), followed by inoculation (6 log_10_ CFU/mL) at pH levels (6.5, 5.5, 4.1, and 3.1). The growth was continuously monitored in 96-well plates using a Stratus plate reader (Cerillo, Charlottesville, VA, USA) under anaerobic conditions.

### Hemolysis activity

The hemolytic activity was assessed using the method described by Nath et al. (69). *B. finegoldii* UO.H1052 was sub-cultured twice and then streaked onto FAA agar supplemented with 5% sheep blood, followed by incubation at 37°C for 72 h. *Staphylococcus aureus* ATCC 25923 was used as a positive control. β-hemolysis was evaluated by monitoring the clear zones surrounding the colonies.

### Cell adhesion and cytotoxicity assays of *Bacteroides finegoldii* UO.H1052 in the Caco-2/HT29 co-culture model

Caco-2 (passage 6) and HT29-MTX (passage 3) cells (ATCC, VA, USA) were cultured in DMEM/High Glucose, 10% fetal bovine serum (FBS), and 1% non-essential amino acids at 37°C in a 5% CO_2_ atmosphere. Cells were grown as a monoculture of Caco-2 and HT29-MTX or as a co-culture composed of 90% Caco-2 and 10% HT29 without antibiotics. Duplicate confluent monolayers or co-cultures were inoculated with *B. finegoldii* UO.H1052 cultures at concentrations of 10^7^–10^8^ CFU/mL and incubated for 2 h at 37°C to allow microbial attachment. After incubation, the wells were washed thrice with PBS to remove non-adherent bacteria. The attached bacteria were detached using 0.1% trypsin, diluted in PBS, and serially diluted to quantify colony-forming units. The adhesion index was defined as the number of bacteria adhering to 100 host cells.

For cytotoxicity assays, Caco-2/HT29-MTX cells (10,000 cells/well) were seeded in 96-well plates and incubated overnight. The medium was replaced with 100 µL of DMEM with different CFS concentrations (0.5%–40%) of *B. finegoldii* UO.H1052. After 24 h of incubation, cell viability and membrane integrity were assessed using LDH assays (CytoTox 96 kit, Promega, Madison, WI, USA), following the manufacturer’s protocols. Cell viability was determined using an ATP assay (CellTiter-Glo; Promega, Madison, WI, USA).

### Modulation of epithelial TEER resistance

Confluent Caco-2/HT29 co-culture monolayers (9:1 ratio of Caco-2 to HT29 cells) were grown on Transwell clear inserts with 0.4 µm pore polyester membranes (Corning, USA) and incubated for 28 days at 37°C in a 5% CO_2_ atmosphere. *B. finegoldii* UO.H1052 CFS was applied to the apical compartment. Two experimental conditions were tested: one exposed to 10 µg/mL LPS from *Serratia marcescens* (Sigma) and the other without LPS. Cell monolayer integrity was assessed by measuring transepithelial electrical resistance over time using an epithelial volt ohm meter (EVOM2; WPI, Germany).

### Serotonin secretagogue capacity *in vitro*

To investigate whether *B. finegoldii* UO.H1052 CFS and EVs could induce serotonin production in RIN14B cells, confluent RIN14B cells (ATCC) were used as a model for serotonergic activity. The cells were cultured in RPMI medium supplemented with 10% FBS at 37°C in 5% CO_2_ and seeded in 24-well plates at a density of 2 × 10^5^ cells/0.5 mL medium. After 72 h, cells were treated with CFS and EVs for 6 h. Following incubation, the RNA and cDNA were prepared as previously described. The expression of *Tph1* and *Mao*a was quantified using RT-qPCR. Primers for *Tph1* and *Maoa* were designed using PrimerQuest Tool from IDT (Table S1).

### Immunomodulatory impact on RAW macrophage

The potential of *B. finegoldii* UO.H1052 CFS and EVs to modulate immune response was assessed on RAW 264.7 mouse macrophage cells (ATCC). Cells were cultured in DMEM/High Glucose containing 10% FBS on T-75 plates, maintained at 37°C in an incubator with 5% CO_2_ until they reached 85% confluence, and seeded in a 96-well plate at a density of 10^4^ cells/well. After 48 h, cells were treated with CFS and EVs in the presence or absence of LPS (5 µg/mL) for 24 h. Cells were lysed in TRIzol, and RNA was extracted using the manufacturer’s recommended protocol. The expression of pro-inflammatory (*Tnfα, Il-1β*, and *Il-6*) and anti-inflammatory (*Il-10 *and* Tgf-β*) cytokines was analyzed by RT-qPCR. cDNA was synthesized using an iSCRIPT cDNA Synthesis Kit and quantified using SYBR Green Master Mix (Bio-Rad) and the primers listed in Table S2.

### Statistical analysis

Data are expressed as mean ± standard error of the mean from a minimum of three experiments. Statistical analyses were conducted using GraphPad Prism version 9.0 (GraphPad Software, San Diego, CA, USA). For comparisons involving multiple groups, two-way ANOVA, followed by Sidak-Bonferroni *post hoc* correction, was performed. Statistical significance was set at *P* ≤ 0.05.

## Data Availability

The 16S rRNA gene sequences and genomic data of *B. finegoldii* UO.H1052 are available from the NCBI database under accession numbers OP690591 and JAQPYU000000000.1, respectively. Metabolomic data were deposited in the EMBL-EBI MetaboLights database using the identifier MTBLS10526.

## SUPPLEMENTAL MATERIAL

Supplemental material aem.00891-25-s0001.pdf Figures S1 to S3; Tables S1 and S2.

ASM does not own the copyrights to Supplemental Material that may be linked to, or accessed through, an article. The authors have granted ASM a non-exclusive, world-wide license to publish the Supplemental Material files. Please contact the corresponding author directly for reuse.
