# Supplementary material for: Serotonergic and immunomodulatory properties of the psychobiotic candidate Bacteroides finegoldii UO.H1052 and its extracellular vesicles
Source: Appl Environ Microbiol. 2025 Aug 21;91(9):e00891-25. doi: 10.1128/aem.00891-25 (PMC12442397; doi:10.1128/aem.00891-25)
Supplement: Supplemental material — Figures S1 to S3; Tables S1 and S2. [file aem.00891-25-s0001.pdf]

Supplementary data:

Serotonergic and immunomodulatory properties of the psychobiotic candidate *Bacteroides finegoldii* UO.H1052 and its extracellular vesicles

Basit Yousuf[1], Galal Ali Esmail[1], Nazila Nazemof[1], Nour Elhouda Bouhlel[1], Zoran Minic[3], and Riadh Hammami[1,5,*]

Fig. S1. Circular plots of the *B. finegoldii* UO.H1052 genome highlighting prophage regions and associated genes identified by PHASTEST (A) and the integrated genomic islands regions indicated by red color mapped along the genome (B). 2

Fig S2. Illustrates quantification of neuroactive metabolites in *B. finegoldii* CFS and EVs. (A-J) Chromatographic peaks and MS1 spectra for GABA, glutamate, tyrosine, tryptophan, and tyramine in CFS and EVs. Metabolite identification was based on retention time and m/z values of pure standard compounds, with a mass tolerance of 5 ppm. Data represent targeted metabolomics analysis using nano-flow LC–MS/MS for quantification. 4

Fig S3. Nanoparticle tracking analysis was used to determine the concentration and diameter of *B. finegoldii* EVs. 5

Table S1. RT-qPCR primer sequences targeting key genes involved in serotonin (5-HT) metabolism used in this study for expression analysis. 6

Table S2. RT-qPCR primer sequences targeting cytokine gene expression analyzed in this study. 6

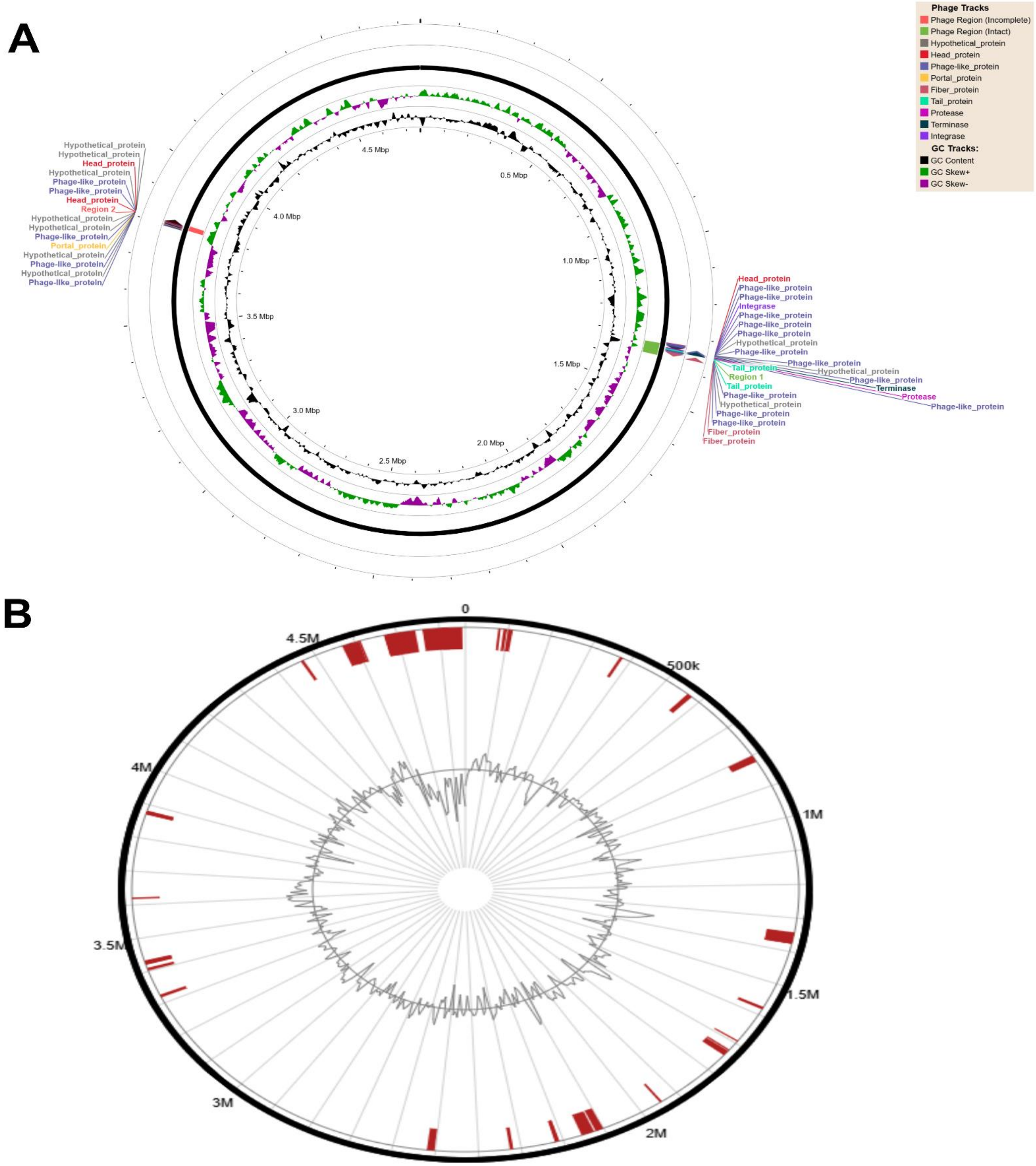

Fig. S1. Circular plots of the *B. finegoldii* UO.H1052 genome highlighting prophage regions and associated genes identified by PHASTEST (A) and the integrated genomic islands regions indicated by red color mapped along the genome (B).

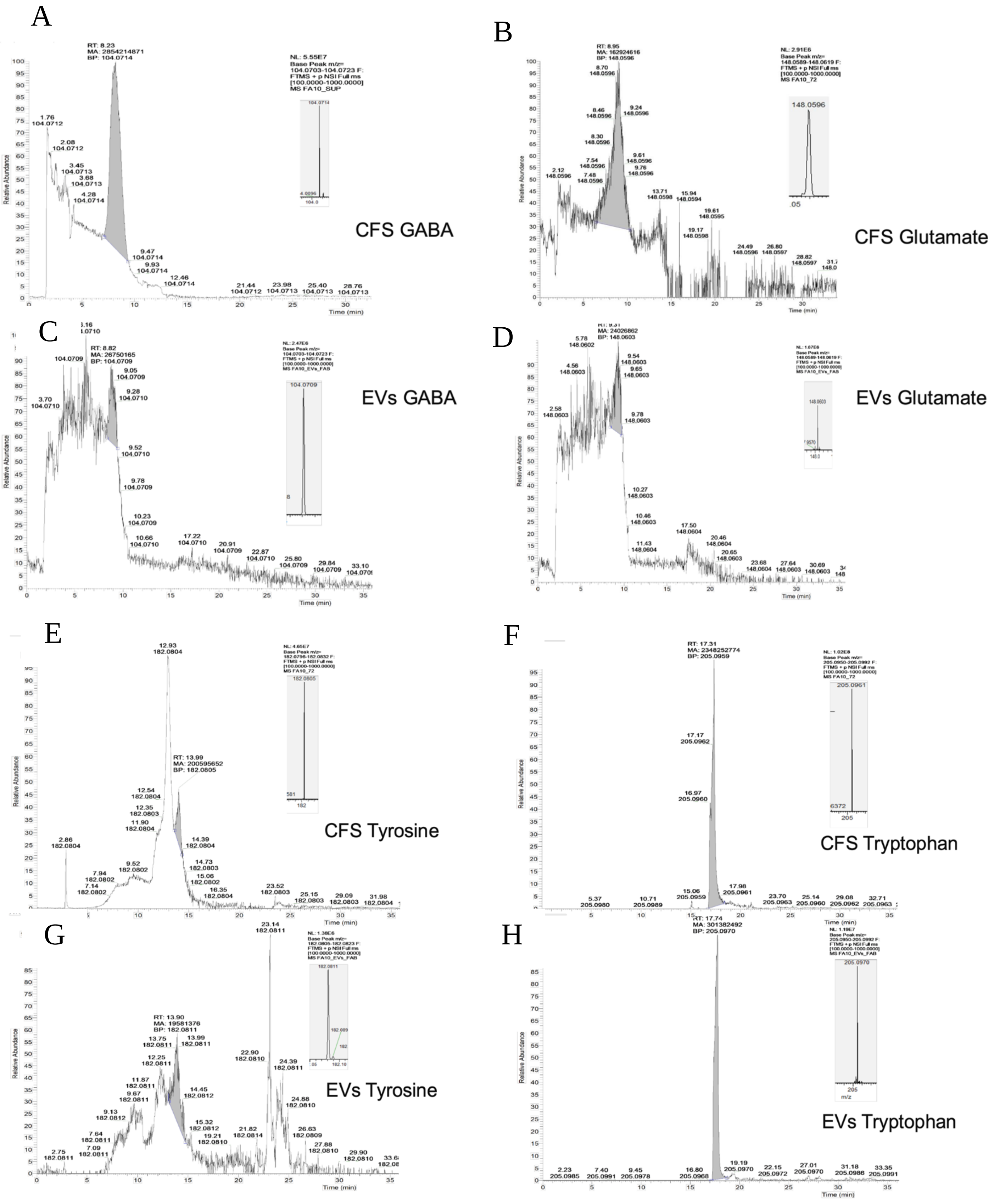
A
CFS GABA
B
CFS Glutamate
C
EVs GABA
D
EVs Glutamate
E
CFS Tyrosine
F
CFS Tryptophan
G
EVs Tyrosine
H
EVs Tryptophan

I

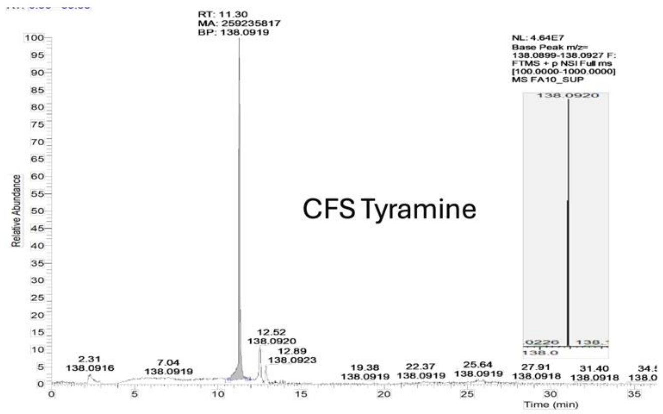

J

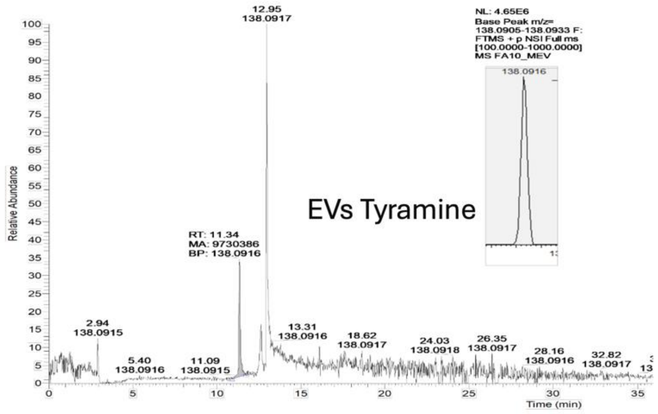

Fig S2. Illustrates quantification of neuroactive metabolites in *B. finegoldii* CFS and EVs. (A-J) Chromatographic peaks and MS1 spectra for GABA, glutamate, tyrosine, tryptophan, and tyramine in CFS and EVs. Metabolite identification was based on retention time and m/z values of pure standard compounds, with a mass tolerance of 5 ppm. Data represent targeted metabolomics analysis using nano-flow LC–MS/MS for quantification.

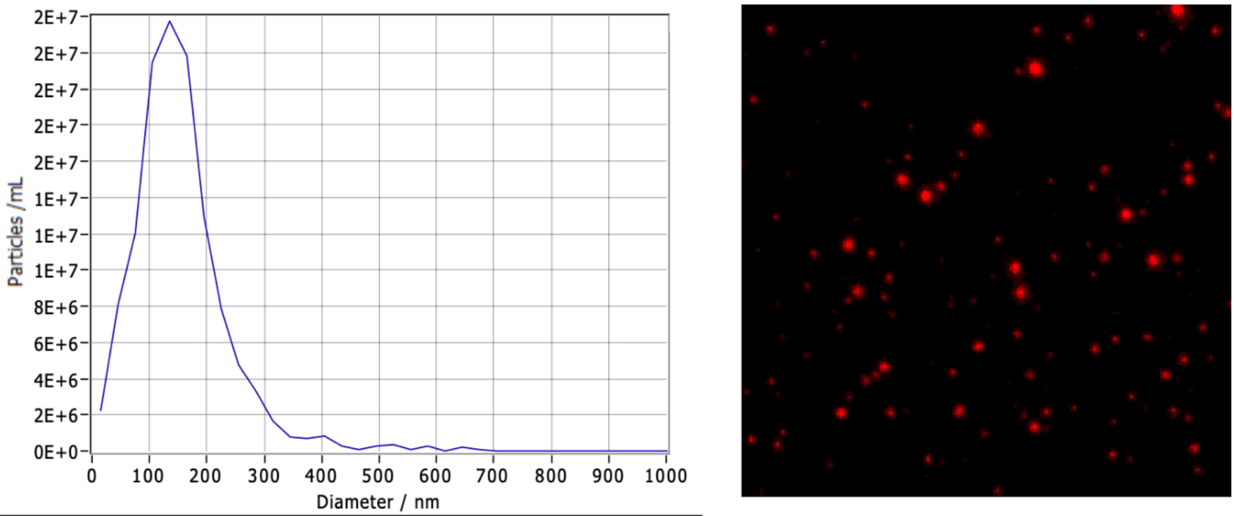

Fig S3. Nanoparticle tracking analysis was used to determine the concentration and diameter of *B. finegoldii* EVs.

Table S1. RT-qPCR primer sequences targeting key genes involved in serotonin (5-HT) metabolism used in this study for expression analysis.

**Primer ID**	**Gene name**	**Sequence**
Tph1_F	Tryptophan Hydroxylase 1	5′-GGCTCTGGTTCTGCGATATT-3′
Tph1_R	Tryptophan Hydroxylase 1	5′-TTGAGTCTGTCCTGGTGTTTG-3′
Maoa_F	Monoamine Oxidase A	5′-CACTGCTCGGATATTCTCAGTT-3′
Maoa_R	Monoamine Oxidase A	5′-GGAGAGCTCAGCTTCACTTTAT-3′

Table S2. RT-qPCR primer sequences targeting cytokine gene expression analyzed in this study.

**Gene**	**Function**	**Sequence**
IL-1β	Pro-inflammatory	5′-TCCCCATGTTGTAGTGACCC-3′
		5′-GGTCTAGAACAGTGGCCCTT-3′
IL-6	Pro-inflammatory	5′-TCCAGTTGCCTTCTTGGGAC-3′
		5′-GTGTAATTAAGCCTCCGACTTG-3′
TNF-α	Pro-inflammatory	5′-AAGGGGATTATGGCTCAGGG-3′
		5′-ACATTCGAGGCTCCAGTGAA-3′
TGF-β1	Anti-inflammatory	5′-AACTTCTGTCTGGGACCCTG-3′
		5′-CTCCGTTTCTCTGTCACCCT-3′
IL-10	Anti-inflammatory	5′-TCACACCCAACCTCTGATCC-3′
		5′-AGGGTCTTCAGCTTCTCACC-3′
β-actin	Housekeeping genes	5′-TGTTACCAACTGGGACGACA-3′
		5′-CTGGGTCATCTTTTCACGGT-3′
GAPDH	Housekeeping genes	5′-GATGCAGTGCCAGGTGAAAATC-3′
		5′-ATCACGTCCTCCATCATCCC-3′
